# Blocking the PI3K/AKT pathway enhances mammalian reovirus replication by repressing IFN-stimulated genes

**DOI:** 10.3389/fmicb.2015.00886

**Published:** 2015-09-02

**Authors:** Jin Tian, Xiaozhan Zhang, Hongxia Wu, Chunguo Liu, Zhijie Li, Xiaoliang Hu, Shuo Su, Lin-Fa Wang, Liandong Qu

**Affiliations:** ^1^State Key Laboratory of Veterinary Biotechnology, Harbin Veterinary Research Institute, Chinese Academy of Agricultural Sciences, Harbin, China; ^2^College of Veterinary Medicine, South China Agricultural University, Guangzhou, China; ^3^Program in Emerging Infectious Diseases, Duke-NUS Graduate Medical School, Singapore, Singapore

**Keywords:** reovirus, PI3K/Akt, endocytosis, enhancing viral replication

## Abstract

Many host cellular signaling pathways were activated and exploited by virus infection for more efficient replication. The PI3K/Akt pathway has recently attracted considerable interest due to its role in regulating virus replication. This study demonstrated for the first time that the mammalian reovirus strains Masked Palm Civet/China/2004 (MPC/04) and Bat/China/2003 (B/03) can induce transient activation of the PI3K/Akt pathway early in infection *in vitro*. When UV-treated, both viruses activated PI3K/Akt signaling, indicating that the virus/receptor interaction was sufficient to activate PI3K/Akt. Reovirus virions can use both clathrin- and caveolae-mediated endocytosis, but only chlorpromazine, a specific inhibitor of clathrin-mediated endocytosis, or siRNA targeting clathrin suppressed Akt phosphorylation. We also identified the upstream molecules of the PI3K pathway. Virus infection induced phosphorylation of focal adhesion kinase (FAK) but not Gab1, and blockage of FAK phosphorylation suppressed Akt phosphorylation. Blockage of PI3K/Akt activation increased virus RNA synthesis and viral yield. We also found that reovirus infection activated the IFN-stimulated response element (ISRE) in an interferon-independent manner and up-regulated IFN-stimulated genes (ISGs) via the PI3K/Akt/EMSY pathway. Suppression of PI3K/Akt activation impaired the induction of ISRE and down-regulated the expression of ISGs. Overexpression of ISG15 and Viperin inhibited virus replication, and knockdown of either enhanced virus replication. Collectively, these results demonstrate that PI3K/Akt activated by mammalian reovirus serves as a pathway for sensing and then inhibiting virus replication/infection.

## Introduction

Reovirus belongs to the family *Reoviridae* and is a non-enveloped, double-stranded RNA virus. They can be isolated from a broad range of avian, mammalian and reptilian hosts (Rosen et al., 1960; Jackson and Muldoon, 1973; Fields et al., 2001). The mammalian orthoreoviruses (MRV) firstly isolated from humans in 1951 commonly infects humans, but is pathogenic only in children (Tyler et al., 2004). Reovirus infection is widespread and 50–100% of adults show seropositivity (Twigger et al., 2012). Most infections result in only mild illness or are asymptomatic. Despite its lack of pathogenicity in humans, recent studies have demonstrated that infection of reoviruses spilled over from wild animals can lead to acute and severe clinical disease in humans (Chua et al., 2007, 2008, 2011; Cheng et al., 2009). These recent discoveries have raised concern about future zoonotic reovirus infections in humans and the need for understanding of reovirus pathogenicity in humans.

Reovirus infection is initiated by interactions between the attachment protein σ1, a cell-surface carbohydrate, and junctional adhesion molecule A (JAM-A; Barton et al., 2001). Next, β1-integrin interacts with the reovirus λ2 protein, which contains both RGD and KGE integrin-binding motifs (Maginnis et al., 2006, 2008), leading to reovirus internalization. Reovirus virions can use both clathrin- and caveolae-mediated endocytic pathways during cell entry (Schulz et al., 2012). It is unknown which host signaling pathways are activated during recognition and endocytosis to provided a favorable cellular environment for viral needs.

The PI3K/Akt signaling pathway is involved in multi-cellular processes such as glucose metabolism, protein synthesis, and proliferation. In addition, the PI3K/Akt pathway appears to be associated with the host cell immune response to counteract viral infection (Diehl and Schaal, 2013). Activation of this pathway contributes to the induction of a set of interferon-stimulated genes (ISGs) through the regulation of the transcriptional repressor EMSY (Ezell and Tsichlis, 2012; Ezell et al., 2012). Nevertheless, a large number of viruses rely on activation of the pathway for their replication. For certain viruses, the PI3K/Akt pathway has been shown to be required not only for viral cell entry but also for subsequent intracellular trafficking and viral replication (Cooray, 2004). Infection with mature vaccinia virus triggers PI3K/Akt signaling, and blocking P13K activation can reduce viral entry in an β1-integrin-dependent manner, suggesting that β1 integrin-mediated PI3K/Akt activation is induced by vaccinia virus (Izmailyan et al., 2012). Inhibition of PI3K signaling decreases HIV infection after viral entry and reverse transcription but prior to HIV gene expression (Francois and Klotman, 2003). Activation of the PI3K/Akt pathway plays an important role in regulating vesicular trafficking and cellular entry of the Zaire Ebola virus (Saeed et al., 2008). The internalization of adenovirus relies on the PI3K-mediated organization of the actin cytoskeleton (Li et al., 1998). Parainfluenza virus 5 (PIV5) and avian leukosis virus (ALV) utilize the PI3K/Akt pathway to enhance synthesis of viral RNAs (Sun et al., 2008; Feng et al., 2011). Activation of the PI3K/Akt pathway by the influenza virus regulates an early step during viral entry, which impairs dimerization of IRF-3 and reduces IRF-3-dependent promoter activity, thus causing the loss or reduction of host antiviral activity (Ehrhardt et al., 2006).

To our knowledge, there has been no study conducted on the relationship between PI3K/Akt signaling pathway and reovirus infection. Here, we report our investigation into the role of PI3K/Akt during reovirus infection *in vitro*. Our results showed that Akt was phosphorylated early during infection with two different reovirus strains in a PI3K-dependent manner. Reovirus virions can use both clathrin- and caveolae-mediated endocytosis, but only the blockage of clathrin-mediated endocytosis suppressed Akt phosphorylation. We also found that activation of PI3K/Akt depended on the focal adhesion kinase (FAK) phosphorylation and not on the receptor tyrosine kinase (RTK) pathway. Inhibition of PI3K activation enhanced reovirus replication by repressing ISG expression, and ISG15 and Viperin played important roles in inhibiting reovirus replication.

## Materials and Methods

### Cells and Viruses

All cell lines were purchased from the China Center for Type Culture Collection (CCTCC). Vero cells and 293T cells were maintained in Dulbecco’s modified Eagle’s medium (DMEM). The A549 human lung-epithelial cell line was cultured in F-12K medium. Mouse L929 cells were maintained in 1640 medium. All media was supplemented with 10% fetal bovine serum and 1% antibiotics, and all the cells were incubated at 37°C in 5% CO_2_.

Bat/China/2003 (B/03) and Masked Palm Civet/China/Liu/2004 (MPC/04) were isolated from healthy animals and propagated in L929 cells. Virus stock was used for subsequent studies. Reovirus serotype I Lang (T1L), reovirus serotype II Jones (T2J) and reovirus serotype III Dear (T3D; ATCC) were propagated in L929 cells.

All virus stocks were purified using CsCl gradients (Smith et al., 1969; Hand and Tamm, 1971) and stored at –80°C for further use.

### Preparation of UV-inactivated Virus

Virus was diluted to 10^7^ PFU/mL in DMEM without serum and irradiated in microtiter plates at a distance of 6 cm by a shortwave (254 nm) UV light for 30 min (Helentjaris and Ehrenfeld, 1977). Loss of infectivity of the UV-irradiated virus was confirmed by a plaque assay. The inability to form plaques were used to confirm UV inactivation (Soares et al., 2009).

### Phylogenetic Analysis of S1 Genes

Phylogenetic analysis and tree construction were based on the nucleotide sequence of the open reading frame of the S1 gene. Phylogenetic analysis of viral S1 genes was conducted by the MEGA 4.0 program (Tamura et al., 2007). Phylogenetic trees were generated by the Neighbor-Joining method using p-distance in MEGA 4.0 software, and 1000 NJ bootstrap replicates were obtained to estimate the phylogenies.

### Serotype Determination

The serotype of B/03 and MPC/04 was identified by hemagglutination inhibition following published procedures (Rosen, 1960). Guinea pigs were infected by instilling 0.1 mL of 10^7^ PFU in each nostril of the anesthetized animal. The antiserum was collected from infected animals 3 weeks later and adsorbed with kaolin (Rosen, 1960). For the HI test, 0.1 mL of 0.85% NaCl containing 4 units of hemagglutinin was added to 0.1 mL of serial twofold dilutions of serum in 0.85% NaCl. The mixtures were shaken briefly and then allowed to stand for 1 h at room temperature before adding 0.2 mL of 1% human type O erythrocytes. The titer of the serum was considered as the dilution that completely inhibited agglutination, and the lowest dilution of each serum was tested. The HI titers are expressed as the highest dilution of serum providing complete inhibition of agglutination. Seropositivity was defined as a HI antibody titer ≥ 1:80.

### Titration

Viral titer determination was done by plaque assay using L929 cells, as previously described (Middleton et al., 2007; Zhang et al., 2015). Briefly, samples were diluted and inoculated into 6-well plates of cells by incubation for 1 h. The monolayers were washed with 2 mL of phosphate-buffered saline (PBS) and covered with 2 mL of serum-free medium 199 (Irvine Scientific) and 1% Bacto Agar (DIFCO) containing 10 μg/mL TLCK-treated α-CHT (Sigma-Aldrich). Plaques were visible 2 to 4 days later, depending on the reovirus strains. Plaques were fixed with 1 mL of 10% paraformaldehyde in PBS for 45 min at room temperature and then the agar overlays were peeled off and the cells were stained with 0.05% crystal violet (Sigma-Aldrich) in 10% paraformaldehyde for 5 min at room temperature followed by two washes with water.

### Virus Infection

To infect the cells, serum-starved A549 cells were washed with PBS and incubated with activated or inactivated virus at the indicated multiplicities of infection (MOI) diluted in F-12K containing 0.2% bovine serum albumin (BSA), 0.1 mg/mL streptomycin and 100 U/mL penicillin at 37°C or 4°C for the indicated times. All media were supplemented with 10% heat-inactivated fetal bovine serum.

For growth of the reovirus in A549 cells, the cells were incubated at 37°C for defined intervals after a 1 h adsorption period at 4°C. Cell supernatants were harvested at the indicated time points and titrated on L929 cell monolayers by the plaque assay.

### Western Blot and ELISA Analyses

At pre-determined time points, the cell monolayers were washed with PBS and lysed. The lysates were collected and were cleared by centrifugation at 10,000 g for 5 min at 4°C. The supernatants for total protein content were determined with a BCA protein assay kit (Beyotime). Total protein (30 μg) was separated by 10% SDS-PAGE and transferred onto nitrocellulose membranes (Millipore). Phosphorylation of EMSY was analyzed by phos-tag SDS-PAGE (Kinoshita et al., 2009). Briefly, the protein was resolved by 8% SDS-PAGE with 100 μM Phos-tag Acrylamide AAL-107 (Wako, Japan) and 10 mM Mncl_2_, and then transferred onto nitrocellulose membranes. Membranes were blocked using 5% skim milk at 37°C for 1 h, then incubated overnight at 4°C with specific rabbit anti-Akt antibody, rabbit anti-phospho-Akt (Ser473) antibody, rabbit anti-glyceraldehyde 3-phosphate dehydrogenase (GADPH) antibody, rabbit anti-phospho-FAK (Tyr397) antibody, rabbit anti-FAK antibody, rabbit anti-phospho-Gab1 (Tyr627) antibody, rabbit anti-Gab1 antibody, rabbit anti-p85α antibody, rabbit anti-Clathrin antibody or rabbit anti-EMSY antibody (Abcam). After three washes in TBST buffer, the membranes were incubated with IRDye 800DX conjugated anti-rabbit IgG or IRDye 800-conjugated anti-mouse IgG (1:8000; Rockland Immunochemicals) diluted in TBST as a secondary antibody at 37°C for 1 h. The membranes were washed three times in TBST, then visualized and analyzed with an Odyssey infrared imaging system (LI-COR Biosciences). The intensities of bands were analyzed with ImageJ 1.49 software. The pictures for analysis were first put together using Adobe Photoshop CS3 software and then the picture was uploaded into ImageJ 1.49 software. The background was subtracted and the intensity of each band was counted. Human IFN-β concentrations in cell culture supernatants were determined using the Human Interferon-β ELISA Kit (BD).

### Inhibitor Treatment

All reagents used in this part of the study were purchased from Sigma. Serum-starved A549 cells were treated with LY294002 (10–50 μM), wortmannin (0.1–5 μM), chlorpromazine (5–50 μM), genistein (50–300 μM), PP2 (30 μM), PP3 (30 μM), or solvent DMSO (0.4%, v/v). The cells were treated for 1 h and then infected as described above. At 30 min post-infection (p.i.), the phosphorylation of Akt, Gab1 and FAK in the lysates was quantified by Western blot analysis.

### Dominant Negative FAK cDNA Construct

Construction of the dominant negative FAK was performed as previously described (Xia et al., 2004). Dominant negative FAK (FAK-DN) was generated by amplification of the FAK cDNA obtained from A549 cells by reverse transcription PCR. *Nhe*I and *BamH*I restriction sites were introduced at the 5′ and 3′ ends of FAK-DN, respectively. FAK-DN was cloned into pcDNA 3.1. The primers used to generate FAK-DN were as follows: fwd, 5′-GCTAGCatgAGCACAATATCGATCAGCAAG-3′; and rev, 5′-GGATCCTCAGTGTGGTCTCGTCTGCCC-3′.

### RNA Analysis

At intervals after the supernatants were removed, total RNA was extracted using the RNeasy Mini Kit (QIAGEN, Dusseldorf, Germany) according to the manufacturer’s protocol. RNA concentrations were determined using a spectrophotometer (260 nm/280 nm). cDNA was generated with a reverse-transcription kit (Takara). Quantitative real-time PCR was used to analyze the expression of GAPDH and the targeted genes with the TaqMan Universal PCR Master Mix kit (Applied Biosystems). After initial denaturation at 94°C for 60 s, amplification was performed over 40 cycles with the following program: denaturation at 94°C for 15 s, primer annealing at 54°C for 30 s, and DNA extension at 72°C for 20 s. The relative quantification of gene expression was analyzed using the 2^–ΔΔCT^ method. The relative expression levels of the targeted gene were normalized to the expression level of the GAPDH gene. The following primers were used: GAPDH_fwd, 5′-TGACCACAGTCCATGCCATC-3′ GAPDH_rev, 5′-GCCAGTGAGCTTCCCGTTCA-3′; Reovirus S4_fwd, 5′-TTGTCGCAATGGAGGTGTGC-3′, Reovirus S4_rev, 5′-TAGACATTGCATGCAGACGA-3′; Akt_fwd, 5′-CACTGTCATCGAACGCACCT-3′, Akt_rev, 5′-ACACCTCCATCTCTTCAGCC-3′; IFITM1_fwd, 5′-TCATCCTGTTACTGGTATTCGGCTC-3′, IFITM1_rev, 5′-GTGGGTATAAACTGCTGTATCTAGGG-3′; ISG15_fwd, 5′-TCCTGGTGAGGAATAACAAGGG-3′, ISG15_rev, 5′-GTCAGCCAGAACAGGTCGTC-3′ and Viperin_fwd, 5′-CAAGACCGGGGAGAATACCTG-3′, Viperin_rev, 5′-GCGAGAATGTCCAAATACTCACC-3′.

### Reporter Plasmids and Luciferase Assays

The pISRE-TA-Luc (Clontech) reporter plasmid was used for monitoring the induction of ISRE-mediated signal transduction pathways. The cells (5 × 10^4^) grown in 48-well plates were transfected with 0.4 μg/well of a reporter plasmid, 0.4 μg/well of an empty plasmid or 0.2 μg/well of a reporter plasmid and 0.2 μg/well of expression plasmid, along with 0.1 μg/well of pRL-TK plasmid (Promega). Cells were infected with reovirus and Sendai virus (SeV, positive control) 12 h after transfection. Luciferase assays were performed 12 h after SeV infection and at indicated time points after reovirus infection. Luciferase activity in these cultures was quantified using the Dual-Luciferase Assay kit (Promega) according to the manufacturer’s instructions.

### IFITM1, ISG15, and Viperin Over-expression and Analysis of Antiviral Effect

IFITM1, ISG15 and Viperin were cloned into p3 × Flag-CMV-10 (Clontech), and the expression of these plasmids was examined by Western blot analysis with an anti-flag antibody. A549 cells (2 × 10^5^) grown in 24-well plates were transfected with 1 μg/well of expression plasmid or an empty plasmid (mock). 24 h after transfection, cells were infected with a MOI of 5. The virus RNA level and viral titer of cell supernatants were determined 24 h after infection.

### Knockdown of p85α, Clathrin, FAK, Akt1, shEMSY, IFITM1, and ISG15

The plasmids expressing shRNAs targeting Akt1 (sc-29196-SH), EMSY (sc-45565-SH), IFITM1 (sc-44549-SH), and ISG15 (sc-43869-SH) as well as the control sequence (sc-108060) were purchased from Santa Cruz Biotechnology. The siRNAs targeting p85α (sc-39125) and clathrin (sc-35068) were obtained from Santa Cruz Biotechnology, and the siRNA targeting FAK (6472s) was ordered from Cell Signaling Technology. The cells were transfected with Lipofectamine 2000 (Life Technologies) according to the manufacturer’s protocol for 48 h. The mRNA and protein levels of p85α, clathrin, FAK, Akt1, IFITM1 and ISG15 were then examined (data not shown).

### The Effect of Akt1 Knockdown on the Growth and Plaque Formation of Reovirus

To analyze the growth of reovirus in A549 cells (2 × 10^5^), each virus at a MOI of 5 was inoculated into cells transfected with 1 μg of shCtrl, 1 μg of shAkt or with no shRNA. After 1 h of viral adsorption at 4°C, the medium was removed, and fresh F-12K medium containing 1% FBS, 100 IU/mL penicillin and 100 μg/mL streptomycin was added to each well. The plate was kept at 37°C. Cell supernatants were harvested at 12, 24, 36, 48, 72, and 96 h post-infection, and viral titers were titrated on L929 cells using the plaque assay.

For the plaque assay, A549 or Vero cells were transfected with 4 μg/well shCtrl or shAkt for 24 h, and the cells were then inoculated with each virus. After incubation for 1 h, the monolayers were covered with 2 ml of 1% Bacto Agar (DIFCO) and serum-free medium 199 (Irvine Scientific) containing 10 μg/ml TLCK-treated α-CHT (Sigma-Aldrich). The plate was photographed 2 to 4 days later to determine the plaque titer.

### Blocking the IFN Signaling Pathway Using Type I and III Receptor Antibodies

The cells (5 × 10^4^) grown in 48-well plates were transfected with 0.4 μg/well of pISRE-TA-Luc reporter plasmid and 0.05 μg/well of pRL-TK plasmid, and then were treated with 10 μg/mL anti-IFNAR1 (ab45172, Abcam) and 20 μg/mL of anti-IFN lambda R1 (ab83865, Abcam) or Cells were infected with Sendai virus 12 h after transfection. Luciferase assays were performed 12 h after SeV infection. Luciferase activity in these cultures was quantified using the Dual-Luciferase Assay kit (Promega) according to the manufacturer’s instructions to analyze the neutralizing capacities of antibodies in an IFN signaling pathway.

### Statistical Analysis

The statistical significance of differences between experimental groups was determined with a paired t test and one-way ANOVA with Prism 5.0 software (GraphPad Software). A *p*-value < 0.05 was selected to indicate significance.

## Results

### Isolation and Preliminary Characterization of Two New Mammalian Reoviruses

After the outbreaks of sever acute respiratory syndrome (SARS) in 2002–2003, epidemiological and pathogen discovery investigation indicated that masked palm civets and bats were most likely the amplifying reservoir hosts for the SARS coronavirus respectively (Kan et al., 2005; Wang et al., 2005; Ge et al., 2013). Since then, other viruses have also been detected or isolated from these two animal groups, including reoviruses (Du et al., 2010; Lelli et al., 2013). The two new mammalian reoviruses used in this study were isolated from bats in 2003 (B/03) and civets in 2004 (MPC/04), respectively. Both were isolated from apparently healthy animals in a surveillance study after the outbreak of SARS.

**FIGURE 1:**
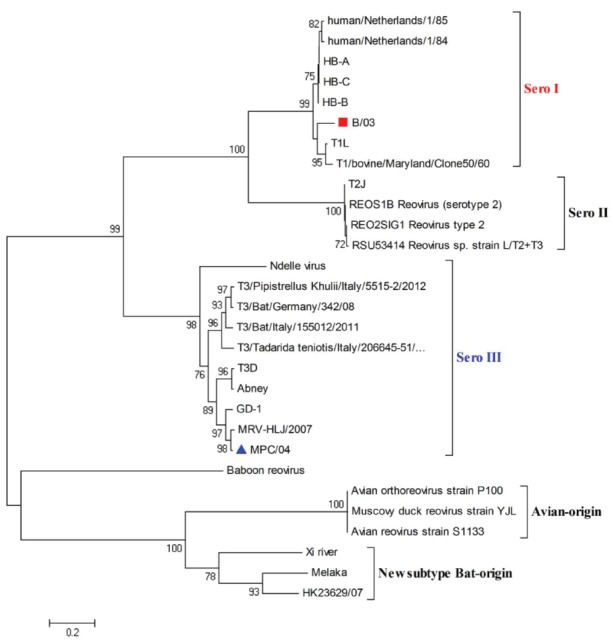
**Phylogenetic analysis of reovirus S1 gene sequences.** The scale bar represents the nucleotide substitutions per site. The numbers above and below the branches indicate bootstrap values. Bootstrap support values > 70 are shown (1000 replicates). ■ (B/03) and ▲ (MPC/04) indicate the two viruses characterized in this study.

Phylogenetic analysis based on the S1 gene revealed that the B/03 strain belonged to serotype I and that the MPC/04 strain belonged to serotype III (Figure 1). This molecular grouping was further confirmed serologically by HI testing (data not shown).

### Infection With B/03 and MPC/04 Reoviruses Transiently Activates Akt in a PI3K-dependent Manner

**FIGURE 2:**
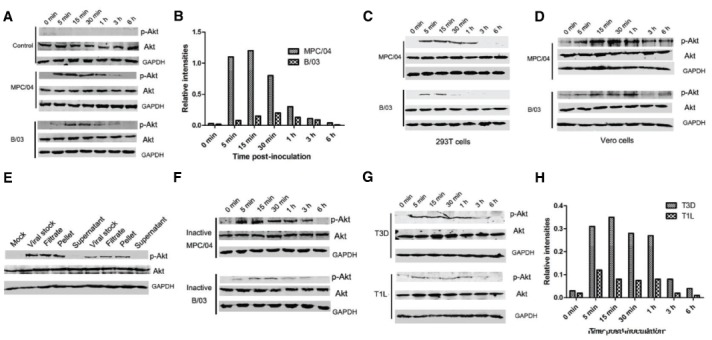
**Determination of Akt phosphorylation in A549 cells.** Serum-starved A549 cells **(A)**, 293T cells **(C)** and Vero cells **(D)** were infected with live MPC/04 and B/03 at a MOI of 5. The lysates from reovirus-infected cells were harvested at the indicated time points. **(E)** Viral stocks were filtered and pelleted. The viral stock, filtrate, pellet and supernatant were used to infect A549 cells. At 30 min p.i., cell lysates were harvested. **(F)** Serum-starved A549 cells were infected with UV-inactivated MPC/04 and B/03. The lysates from reovirus-infected cells were harvested at the indicated time points. **(H)** Serum-starved A549 cells were infected with T1L and T3D at a MOI of 5. In all assays, phosphorylated Akt (Ser473) was detected by Western blotting. Equal protein loading was verified using total Akt and GAPDH on the same membranes. **(B,H)** Quantification of relative p-Akt band intensities to Akt in **(A)** and **(G)**. The results were confirmed in three independent experiments.

Because of the biological importance of the PI3K/Akt pathway in viral infection, we investigated whether B/03 and MPC/04 infections lead to activation of this pathway. In contrast to mock-treated cells, infection with both viruses resulted in a rapid increase in Akt phosphorylation within 5–15 min post-infection (p.i.), a subsequent decline by 30 min p.i. and a return to background levels by 6 h p.i. (Figure 2A). Akt phosphorylation in MPC/04-infected cells began after approximately 5 min, which was faster than the time required (approximately 15 min) in B/03-infected cells (Figure 2A). Moreover, Akt phosphorylation in MPC/04-infected cells at each time point reached higher levels than in B/03-infected cells (Figure 2B). Similar results were obtained in 293T cells (Figure 2C) and Vero cells (Figure 2D). To eliminate any secreted factors that could possibly have triggered production of phosphorylated Akt (p-Akt), viral stocks were filtered and pelleted by centrifugation, and they were then resuspended and inoculated into cells. As shown in Figure 2E, both viral filtrate and pellet from viral stocks triggered p-Akt at 30 min p.i. In contrast, no signal was detected in cellular extraction from L929 cells inoculated with supernatant after centrifugation. To demonstrate further that Akt phosphorylation depended on viral infection, both viruses were exposed to shortwave (254 nm) UV light treatment, and virus inactivation was verified by plaque assay. When inactivated viruses were inoculated into A549 cells, phosphorylation of Akt was still detected (Figure 2F). These results demonstrated that MPC/04 and B/03 reovirus particles were sufficient to activate Akt in the early stage of cell infection.

To analyze whether the infection with T1L and T3D leads to different results than those from B/03 and MPC/04, the levels of p-Akt in A549 cells were determined. As shown in Figure 2G, Akt phosphorylation was detected in the cells infected with both viruses from 5 min to 1 h p.i., and the levels of p-Akt in T3D-infected cells were higher than those in T1L-infected cells (Figure 2H).

**FIGURE 3:**
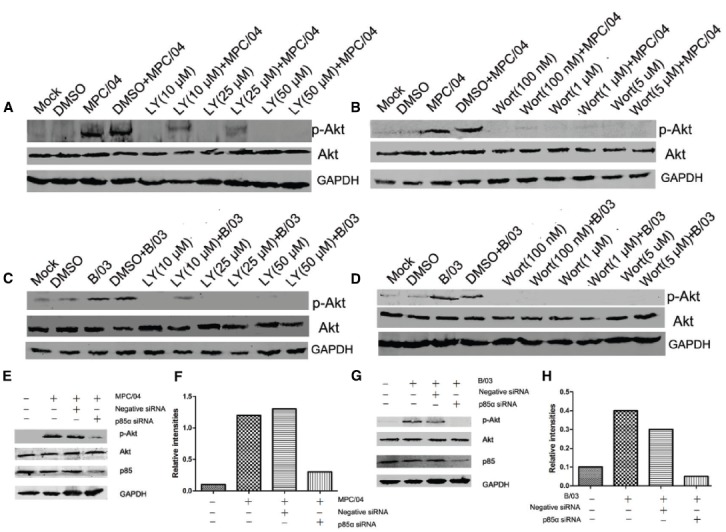
**Role of PI3K in Akt phosphorylation.** Serum-starved A549 cells were pre-incubated with LY294002 (LY; 10–50 μM), wortmannin (Wort; 100 nM–1 μM) or DMSO (0.4%, v/v) for 1 h and subsequently infected with MPC/04 **(A,B)** or B/03 **(C,D)** at a MOI of 5. A mock-infected lane was included in each panel as a negative control. **(E,G)** A549 cells were transfected with 15 pmol/well negative control (Negative siRNA) or p85α siRNA. 24 h after transfection, cells were infected with MPC/04 **(E)** or B/03 **(G)** at a MOI of 5. Cell lysates were collected at 30 min p.i. and separated by SDS-PAGE. In all assays, phosphorylated Akt (Ser473) was detected by Western blotting. Equal protein loading was verified using total Akt and GAPDH on the same membranes. The levels of p85α were determined by Western blot analysis using an anti-p85α antibody. **(F,H)** Quantification of relative p-Akt band intensities to Akt in **(E)** and **(G)**. The results were confirmed in three independent experiments.

To determine whether Akt phosphorylation was PI3K-dependent, the role of PI3K in Akt phosphorylation following reovirus infection was investigated using the specific PI3K inhibitors, LY294002 (LY) and wortmannin (Wort), or siRNA targeting the PI3K p85α subunit. As expected, pretreatment with LY (50 μM) or Wort (0.1 μM) completely inhibited reovirus infection-induced Akt phosphorylation (Figures 3A–D). Knockdown of p85α reduced the level of p-Akt at 30 min p.i. (Figures 3E–H), indicating that the activation of Akt induced by reovirus infection depended on PI3K.

### Activation of PI3K/Akt is Associated With Clathrin-mediated Endocytosis

**FIGURE 4:**
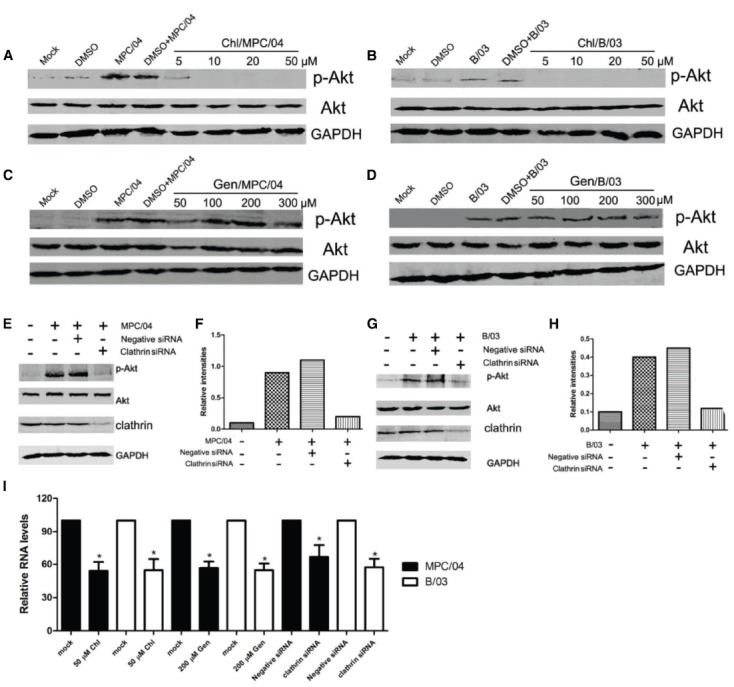
**Role of clathrin-mediated endocytosis in the activation of PI3K/Akt early in infection.** Serum-starved A549 cells were pretreated with various concentrations of chlorpromazine (Chl; 5–50 μM) and genistein (Gen; 50–300 μM) for 30 min, and the cells were then infected with MPC/04 **(A,C)** and B/03 **(B,D)** at a MOI of 5. A mock-infected lane was included in each panel as a negative control. After 30 min p.i., the cell lysates were collected and separated by SDS-PAGE. The levels of p-Akt (Ser473), total Akt and GAPDH were determined by Western blot analysis as detailed in Methods. **(E,G)** A549 cells were transfected with 15 pmol/well of negative control (Negative siRNA) or clathrin siRNA. 24 h after transfection, cells were infected with MPC/04 **(E)** or B/03 **(G)** at a MOI of 5. After 30 min, the cell lysates were collected and separated by SDS-PAGE. The levels of p-Akt (Ser473), total Akt, clathrin and GAPDH were determined by Western blot analysis. **(F,H)** Quantification of relative p-Akt band intensities to Akt in **(E)** and **(G)**. The results were confirmed in three independent experiments. **(I)** A549 cells pretreated with 50 μM Chl or 200 μM Gen for 1 h or transfected with 15 pmol/well of negative control (Negative siRNA) or clathrin siRNA for 12 h were infected with MPC/04 or B/03 at a MOI of 5. Viral RNA levels were determined at 24 h post infection. The data shown in I represent the results of three independent experiments. *p < 0.05. Error bars indicate SD.

Because Akt phosphorylation occurs within 5–15 min p.i., it is possible that cellular endocytosis play key roles in the activation of PI3K/Akt. Thus, we examined whether PI3K/Akt activation depends on cellular endocytosis. Chlorpromazine (Chl), an inhibitor of clathrin-mediated endocytosis, completely suppressed Akt phosphorylation at a concentration of 10–50 μM (Figures 4A,B) and inhibited reovirus RNA production (Figure 4I). Genistein (Gen), an inhibitor of caveolar endocytosis, did not decrease the levels of p-Akt (Figures 4C,D) at a concentration of 300 μM, but it did inhibit the caveolar pathway and reovirus replication in A549 cells at 200 μM (Figure 4I). To confirm that clathrin-mediated endocytosis is required for the induction of PI3K/Akt, cells were transfected with siRNA targeting clathrin. Knockdown of clathrin reduced the levels of p-Akt (Figures 4E–H) and reduced reovirus RNA production (Figure 4I).

### Activation of PI3K/Akt Depends on FAK Phosphorylation

**FIGURE 5:**
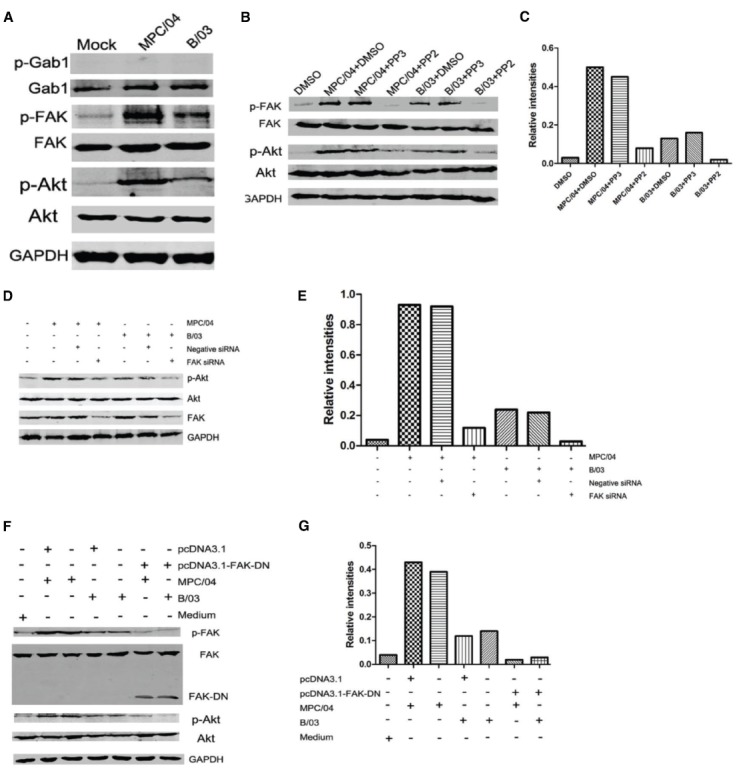
**Dependence of PI3K/Akt activation on FAK phosphorylation. (A)** Serum-starved A549 cells were infected with MPC/04 and B/03 at a MOI of 5 or mock-infected for 30 min. The cell lysates were collected, and the expression of Gab1, phosphorylated Gab1 (Tyr627; p-Gab1), FAK, phosphorylated FAK (Tyr397; p-FAK), p-Akt (Ser473), total Akt and GAPDH was determined by Western blot analysis. **(B)** Serum-starved A549 cells were pretreated with 10 μM PP2 or PP3 (as control) for 60 min and then infected with MPC/04 and B/03 at a MOI of 10 or mock-infected for 30 min. The cell lysates were collected, and the expression of p-FAK, FAK, p-Akt (Ser473), Akt and GAPDH was determined as for A. **(D)** A549 cells were transfected with 15 pmol/well of negative control (Negative siRNA) or FAK siRNA. 24 h after transfection, cells were infected with MPC/04 or B/03 at a MOI of 5. After 30 min, the cell lysates were collected, and the levels of p-Akt (Ser473), total Akt, FAK and GAPDH were determined by Western blot analysis. **(F)** A549 cells were transfected with 2 μg/well of pcDNA3.1, pcDNA3.1-FAK-DN or medium for 24 h, and the cells were then infected with MPC/04 and B/03 at a MOI of 5 or mock-infected for 30 min. The p-FAK, FAK, p-Akt (Ser473), Akt and GAPDH levels were determined as for **(A)**. The results shown in **(A–C)** were confirmed in three independent experiments. **(E,G)** Quantification of relative p-Akt band intensities to Akt in **(E)** and **(G)**. The results were confirmed in three independent experiments.

FAK is upstream of PI3K/Akt in the β1-integrin pathway (Xia et al., 2004). Blocking FAK by pharmacologic inhibition or by dominant negative FAK attenuates phosphorylation of the p85 subunit of PI3K and Akt (Xia et al., 2004). Grb2-associated binder 1 (Gab1) is an adapter protein, and it can bind to growth factor receptor-binding protein 2 (Grb2). Both Gab1 and Grb2 act downstream of RTK (Nishida and Hirano, 2003). Phosphorylated Gab1 interacts with Grb2, SHP2 and the p85 subunit of PI3K (Nishida and Hirano, 2003). First, we examined the phosphorylation of FAK and Gab1 in reovirus infection. At 30 min p.i., infection with either B/03 or MPC/04 led to phosphorylation of Akt and FAK but not Gab1 (Figure 5A). PP2, a selective Src-kinase family inhibitor that blocks integrin-induced FAK phosphorylation (Xia et al., 2004), abolished the phosphorylation of tyrosine 397 of FAK and serine 473 of Akt with both B/03 and MPC/04 infection (Figures 5B,C). In contrast, PP3, an inactive analog of PP2, had no effect on FAK or Akt phosphorylation (Figures 5B,C). To eliminate the potential non-specificity issue caused by pharmacological inhibitors, we knocked down the expression of FAK by siRNA and found that p-Akt levels were reduced (Figures 5D,E). Furthermore, when cells were transfected with the dominant negative FAK (FAD-DN) plasmid, phosphorylation of FAK and Akt was blocked in response to infection of both viruses (Figures 5F,G). These results suggested that the activation of PI3K/Akt signaling was mediated by FAK during B/03 or MPC/04 infection.

### PI3K/Akt Pathway Regulates Viral Replication

**FIGURE 6:**
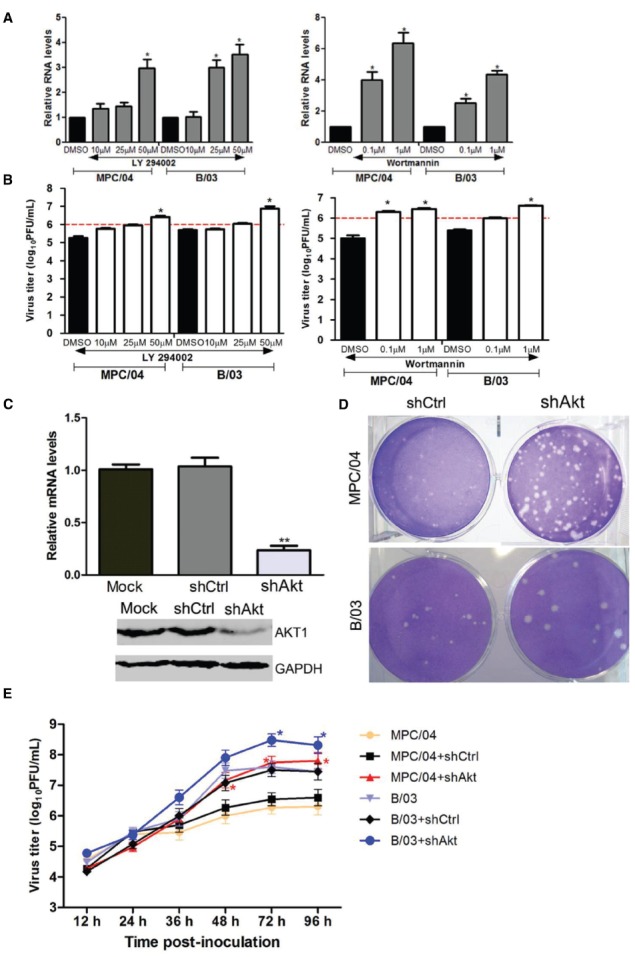
**Modulation of reovirus replication by the PI3K/Akt pathway.** Serum-starved A549 cells were pre-incubated with increasing concentrations of LY294002, wortmannin or DMSO for 1 h, and they were then infected with MPC/04 and B/03 at a MOI of 5 in the presence of each drug for 24 h. The total RNA of the cells was prepared, and S4 gene expression was analyzed by qPCR **(A)**. Cells and cell supernatants were harvested together, and subjected to a single freeze/thaw step for analysis of viral titers **(B)**. Expression of Akt1 was determined by qPCR analysis and Western blot analysis using Akt1- and GAPDH-specific antibodies **(C)**. Effect of inhibition of Akt1 expression on virus replication was also evaluated by plaque sizes in A549 cells **(D)**. A549 cells were transfected with 4 μg/well of shAkt or shCtrl for 24 h, and the cells were then infected with MPC/04 and B/03. After 1 h incubation, the monolayers were covered with 2 ml of 1% Bacto Agar and 2 × MEM containing 10 μg/ml TLCK-treated α-CHT. Plaques were photographed 4 days later. **(E)** A549 cells were transfected with 2 μg/well of shAkt or shCtrl for 12 h, and the cells were then infected with MPC/04 or B/03 at a MOI of 5. Cell supernatants were harvested at the indicated time point and titrated on L929 cells for the plaque assay. The data shown in **(A–C)** and **(E)** represent the results of three independent experiments. *p < 0.05 and **p < 0.001. Error bars indicate SD.

Our previous study showed that activated PI3K/Akt was required for reovirus MPC/04 replication (Zhang et al., 2015). To investigate whether PI3K/Akt signaling was involved in both MPC/04 and B/03 infection, both viral RNA transcription and viral titers were measured after PI3K inhibition. The real-time RT-PCR results showed that pretreatment with LY294002 significantly increased MPC/04 RNA synthesis at a concentration of 50 μM (*p* < 0.05) and B/03 RNA synthesis at a concentration of 25 μM (*p* < 0.05; Figure 6A). Pretreatment with wortmannin at all tested concentrations led to a 3- to 5-fold increase (*p* < 0.05) in MPC/04 RNA and a 1- to 4-fold increase (*p* < 0.05) in B/03 RNA (Figure 6A). The viral titer results were in accordance with the results for RNA (Figure 6B). The growth of MPC/04 and B/03 in A549 cells pretreated with LY294002 (50 μM) increased 5.5- and 5-fold, respectively (*p* < 0.05; Figure 6B left), and wortmannin (1 μM) pretreatment led to increases of 7- and 4.4-fold, respectively (*p* < 0.05; Figure 6B right).

To further confirm these results, RNA interference (RNAi) technology was employed to specifically reduce the expression of Akt1. Inhibition of Akt1 expression in A549 cells was confirmed by both qPCR and Western blot analyses (Figure 6C). A549 cells were transfected with shRNA control (shCtrl) and Akt shRNA (shAkt) constructs. After infection with MPC/04 and B/03, the plaques for A549-shCtrl and A549-shAkt were analyzed. As shown in Figure 6D, the plaques in infected A549-shAkt cells were larger than the plaques in A549-shCtrl cells. The growth of MPC/04 and B/03 in cells transfected with shAkt was compared with that in mock-transfected cells (Figure 6E). The results showed that knockdown of Akt promoted the replication of both viruses at 72 and 96 h p.i.

### PI3K/AKT Inhibits Viral Replication Via Increasing IFN-stimulated Genes Expression

**FIGURE 7:**
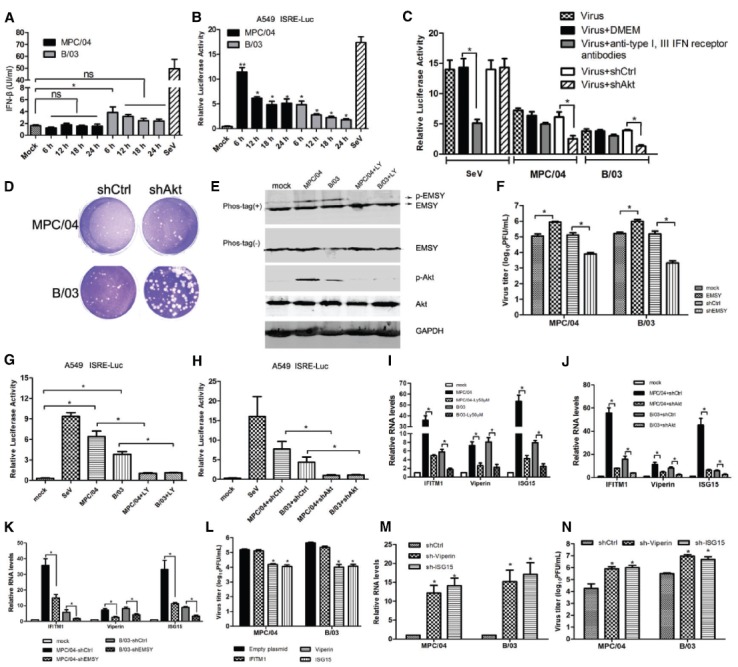
**Role of IFN-stimulated genes in PI3K/AKT-mediated modulation of virus replication. (A)** A549 cells were infected with MPC/04 and B/03 at a MOI of 5, and cell supernatants were collected at indicated time points for analysis of the concentration of IFN-β by ELISA. **(B)** A549 cells were co-transfected with pISRE-TA-Luc and RLTK, and the cells were then infected with MPC/04 and B/03 at a MOI of 5 at 12 h post-transfection. Relative luciferase activities were determined at different time points after reovirus infection using a luciferase assay kit. Sendai virus (SeV) infection was included as a positive control, and its relative luciferase activity was determined at 12 h post-infection. Transfection but no stimulation served as a mock control. **(C)** A549 cells were co-transfected with pISRE-TA-Luc and RLTK or shAkt or shCtrl, and the cells were treated with anti-IFNAR1 and -IFN lambda R1 antibodies or without antibodies 12 h after transfection. 2 h after treatment, cells were infected with MPC/04 and B/03 at a MOI of 5 or SeV. Relative luciferase activities were determined at 24 h post infection using a luciferase assay kit. **(D)** Vero cells were transfected with 4 μg/well of shAkt, transfected with 4 μg/well of shCtrl or left untransfected for 24 h, and the cells were then infected with MPC/04 and B/03. After a 1 h incubation, the monolayers were covered with 2 ml of 1% Bacto Agar and 2 × MEM containing 10 μg/ml TLCK-treated α-CHT. Plates were photographed 4 days later for plaque counting. **(E)** Analysis of phosphorylated EMSY in MPC/04- and B/03-infected cells with and without LY294002 (50 μM) treatment at 30 min post-infection. **(F)** A549 cells were transfected with empty plasmid or a plasmid expressing EMSY, EMSY-targeting shRNA or control (shCtl). 24 h after transfection, the cells were infected with MPC/04 and B/03 at a MOI of 5, and viral titers of cell supernatants were determined 24 h after infection. **(G)** 12 h after A549 cells were cotransfected with pISRE-TA-Luc and RLTK, the cells pretreated with or without LY294002 (50 μM) were infected with MPC/04 and B/03 at a MOI of 5 or mock infected at 12 h post-transfection. SeV infection was included as a positive control. Relative luciferase activities were determined at 12 h p.i. using a luciferase assay kit. **(H)** A549 cells were cotransfected with pISRE-TA-Luc, RLTK and shAkt or shCtrl (negative control). 12 h after transfection, the cells were infected with MPC/04 and B/03 at a MOI of 5 or mock infected at 12 h post-transfection. SeV infection was included as a positive control. Relative luciferase activities were determined at 12 h p.i. using a luciferase assay kit. **(I–K)** Analysis of mRNA levels of IFITM1, ISG15 and Viperin in MPC/04- and B/03- infected cells with or without pre-treatment with LY294002 (50 μM) **(I)** or with transfection with shAkt or shCtrl (negative control) **(J)** or with transfection with shEMSY or shCtrl (negative control) **(K)** by qPCR. **(L–N)** 24 h after transfection with plasmids expressing IFITM1, ISG15, Viperin **(L)**, shCtl, ISG15-targeting shRNA (sh-ISG15) or Viperin-targeting shRNA (sh-Viperin) **(M,N)**, cells were infected with MPC/04 and B/03 at a MOI of 5, and the relative RNA levels of viral S4 gene **(M)** and viral titers of cell supernatants **(L,N)** were determined 24 h after infection. Data represent the results of three independent experiments. *p < 0.05 and **p < 0.001. Error bars indicate SD.

To explore the mechanism of PI3K/Akt regulation of reovirus replication, we analyzed the role of apoptosis and interferon in reovirus replication. Akt promotes cell survival by inhibiting apoptosis via phosphorylating and inactivating multiple targets, including Bad (7), forkhead transcription factors (8), c-Raf (9) and caspase-9(Cardone et al., 1998; Bonni et al., 1999; Zimmermann and Moelling, 1999). Suppression of Akt activation should promote cell death and may increase the release of virus particles. Treatment with TRAIL did not affect reovirus replication in A549 cells (data not shown). The levels of interferon production in infected cells were analyzed. MPC/04 infection did not significantly increase the production of IFN-β (Figure 7A). B/03 infection increased the production of IFN-β only at 6 h p.i. (Figure 7A), but this increase did not occur at the other time points (Figure 7A). However, activation of the IFN-stimulated response element (ISRE) was detected during infection of both viruses (Figure 7B). To rule out the effect of endogenous interferons, activation of the ISRE was examined in A549 cells treated with anti-type I and III receptors antibodies before reovirus infection. Both viruses triggered the ISRE (Figure 7C), and downregulation of Akt by RNAi nearly abolished activation of the ISRE (Figure 7C). The plaque assays in Vero cells revealed that knockdown of Akt formed larger plaques than mock-transfected cells (Figure 7D), suggesting that suppression of Akt increases the cytopathic effect of both reoviruses. These results demonstrated that reovirus infection-mediated induction of ISRE was independent of IFN production.

It has been shown that the activated PI3K/Akt-dependent pathway contributes to the induction of a set of ISGs through the regulation of the EMSY transcriptional repressor (Ezell and Tsichlis, 2012; Ezell et al., 2012). Our data (Figure 7E) demonstrated that infection of MPC/04 or B/03 also resulted in the phosphorylation of EMSY, and LY294002 (50 μM) treatment inhibited the phosphorylation. Overexpression of EMSY repressed the induction of ISRE (data not shown) and promoted viral replication (Figure 7F), and downregulation of EMSY by RNAi promoted the induction of ISRE (data not shown) and inhibited viral replication (Figure 7F). Infection with both viruses increased the relative luciferase activity via the ISRE regulatory element in luciferase assays, and both LY294002 (50 μM) treatment (Figure 7G) or knockdown of Akt (Figure 7H) eliminated virus-induced activation of ISRE, indicating that activated PI3K/Akt in reovirus infection triggered the ISRE via EMSY. Additionally, as shown in Figures 7I–K, both infections significantly increased the transcription of IFITM1, ISG15 and Viperin, and a 50 μM LY294002 treatment (Figure 7I) or knockdown of Akt (Figure 7J) or knockdown of EMSY (Figure 7K) reduced the mRNA levels of IFITM1, ISG15, and Viperin. More importantly, overexpression of ISG15 and Viperin decreased viral titers (Figure 7L), and knockdown of ISG15 and Viperin increased viral RNA levels (Figure 7M) and viral titers (Figure 7N).

These data suggested that the activated PI3K/Akt pathway contributes to the production of a set of ISGs through the EMSY transcriptional repressor, which inhibits the replication of reovirus.

## Discussion

In this study, we demonstrated the role of the PI3K/Akt signaling pathway in regulating reovirus infection. We showed that Akt is phosphorylated early in MPC/04 and B/03 infection in a PI3K-dependent manner and that Akt phosphorylation is triggered and/or mediated by clathrin-mediated endocytosis. We also demonstrated that activation of PI3K/Akt depends on β1-integrin-mediated phosphorylation of FAK. Importantly, we also found that inhibition of PI3K activation increases viral RNA synthesis and viral yield, and we showed that PI3K/Akt activation is an important determinant for the difference observed in growth between MPC/04 and B/03. Finally, we found that PI3K/AKT inhibits viral replication via increasing IFN-stimulated gene expression. These results confirmed that PI3K/Akt signaling regulates reovirus replication and that activation of PI3K/Akt signaling is an important antiviral mechanism.

Many wildlife species are reservoirs of some important pathogens [for example, human immunodeficiency virus (HIV) and Ebola virus] that threaten the health of humans. SARS CoV-like viruses have been isolated from horseshoe bats (Li et al., 2005; Wang and Eaton, 2007; Ge et al., 2013) and palm civets (Guan et al., 2003). The viruses used in this study were isolated from bat and palm civets. It is well documented that bat-borne reoviruses can be transmitted to and cause clinical diseases in humans (Chua et al., 2007). Understanding the mechanisms of infection and pathogenesis by viruses of wildlife origin will be essential for the prevention and control of infectious disease emergence.

Field viruses typically undergo mutation upon adaptation to cell culture. The changes may be involved in virus attachment or evasion of innate immunity. During the process of propagation on L929 cells, no mutation in amino acid sequence was detected between the genomes from the first passage virus and the second and third passage viruses. Reovirus is isolated from the respiratory and enteric tracts of humans and animals (Gauvin et al., 2013). Both MPC/04 and B/03 were isolated from lung tissues and replicated in same tissues in mice (unpublished data). A549 cells as a cell model for investigating the pathogenic mechanism and characteristics of some respiratory viruses were used in this study and could represent natural environment for infection of reovirus.

Herpesvirus binding to α3β1 integrin (Akula et al., 2002) and cytomegalovirus binding to β1-integrin (Feire et al., 2004) activate FAK. In addition, adenovirus binding of α_*v*_ integrins induces activation of PI3K, which is required for adenovirus endocytosis (Li et al., 1998). Because of their wide distribution, integrins are common receptors of diverse viral pathogens (Izmailyan et al., 2012). Reovirus infection is initiated by interactions between the attachment protein σ1 and JAM-A. Blockage of β-integrin by a specific antibody inhibits reovirus infection in HeLa cells, and expression of the β1-integrin and JAM-A in non-permissive chicken embryo fibroblasts confers susceptibility to reovirus infection (Maginnis et al., 2006). These results suggest that β1-integrin can affect reovirus infection. PI3K/Akt can be activated by both integrin- and RTK-mediated endocytosis pathways (Izmailyan et al., 2012; Goh and Sorkin, 2013). Activation of both pathways can lead to clathrin-mediated endocytosis. In this study, we found that the activation of PI3K/Akt depended on the upstream molecule FAK and clathrin-mediated endocytosis during reovirus infection. Further investigation is required to understand whether reovirus binds to β1-integrin and lead to the phosphorylation of FAK and activation of PI3K/Akt.

The initial timing of activation of PI3K/Akt depends on the virus strain. Activation of PI3K/Akt can be initiated early in infection or replication. Exogenous ALV infection triggers PI3K/Akt signaling in the early stage (Feng et al., 2011), and this pathway can be suppressed by an inhibitor specific for clathrin-mediated endocytosis. β1-integrin-mediated PI3K/Akt activation is induced by vaccinia virus at 20 min p.i. (Izmailyan et al., 2012). For influenza A virus infection, PI3K/Akt activation occurs at the late stage, which depends on NS1 binding to p85β (Ehrhardt et al., 2007). In this study, MPC/04 and B/03 infection activated PI3K/Akt in the early stage of infection, and blockage of PI3K/Akt activation did not affect reovirus absorption and entry (data not shown). Blockage of PI3K/Akt during infection by ALV, vaccinia virus, and other viruses inhibits viral replication, but in our case, this blockage increased viral RNA synthesis and viral yield of MPC/04 and B/03 during infection. Thus, PI3K/Akt plays an important role in recognizing reovirus infection in the early stage and inhibiting virus replication.

In this study, we found that there may be some correlation between PI3K/Akt activation and viral pathogenesis. PI3K/Akt activation inhibited reovirus replication via up-regulating some of the ISGs. MPC/04 infection stimulated more robust phosphorylation of Akt (Figure 2) and led to higher ISGs expression (Figure 7I) compared to B/03 and the results from Figure 6E showed that MPC/04 replicate much less than B/03. More importantly, B/03 is more pathogenic than MPC/04 in mice (unpublished data) and reovirus T1L showed higher virulence than T3D in mice (Gauvin et al., 2013). These data indicated that PI3K/Akt may be associated with different virulence among different reovirus isolates. However, more evidence needs to be identified.

The precise molecular mechanism(s) for regulating viral replication by PI3K/Akt are not yet fully elucidated. Interferon-stimulated transcription is thought to occur mainly through the action of the JAK/STAT pathway. Recent findings have demonstrated that activation of the PI3K/Akt pathway contributes to the induction of a set of ISGs through regulating the EMSY transcriptional repressor (Ezell and Tsichlis, 2012). In this study, we found that ISRE was activated in an interferon-independent, PI3K/Akt-dependent manner. In addition, the phosphorylation of EMSY was induced via PI3K/Akt after infection, and EMSY may regulate the induction of ISRE and reovirus replication. Further, reovirus infection up-regulated the transcription of ISGs, namely IFITM1, ISG15 and Viperin, and blockage of the PI3K/Akt pathway in reovirus infection inhibited the expression of IFITM1, ISG15 and Viperin. Overexpression of ISG15 and Viperin decreased reovirus replication, and knockdown of either enhanced reovirus replication. Although interferons are potent antiviral factors, many viruses, in turn, have evolved multiple strategies to counteract the IFN system (Didcock et al., 1999; Wang et al., 2000, 2010, 2011; Fensterl et al., 2005; de Los Santos et al., 2006). We found that reovirus infection did not induce a high production of IFN-β, which may inhibit antiviral effects mediated by the IFN-β response. As a result of reovirus infection, activated PI3K/Akt promoted the induction of ISRE and increased the expression of ISGs, which can function as a compensatory pathway in interferon signaling to inhibit reovirus replication.

Zhang et al. (2015) reported that PI3K/Akt/p53 signaling pathway was activated upon reovirus strain MPC/04 infection and the activation suppressed the viral replication. In this study, activated PI3K/Akt pathway suppresses reovirus replication through EMSY phosphorylation and ISG induction. Both studies found that PI3K/Akt was involved in reovirus infectin, but provided different pathways. EMSY and p53 are known to involving DNA damage/repair pathways (Cousineau and Belmaaza, 2011), and any cross-talk between two results may exist. However, we found that overexpression of p53 promoted reovirus replication, but did not affect the expression of ISGs (data not shown). So, both may affect reovirus replication via different pathways.

In conclusion, our study revealed, for the first time, that the PI3K/Akt pathway plays an important part in regulating reovirus replication and presumably in pathogenesis. This new finding will not only help us to better understand the role of the PI3K/Akt pathway in the general virus-host interactions, but it will also be important for future investigations of the mechanisms of reovirus-host specificity and pathogenesis as well as for the development of novel prevention and control strategies for reovirus infection in human and animals.

## Author Contributions

JT conceived the study and wrote the paper. XZ, HW, and CL designed, performed and analyzed all the experiments. ZL and XH provided technical assistance and prepared all the figures. LQ, LW, and SS designed the study and revised the manuscript. All authors reviewed the results and approved the final version of the manuscript.

### Conflict of Interest Statement

The authors declare that the research was conducted in the absence of any commercial or financial relationships that could be construed as a potential conflict of interest.
